# 

**Published:** 1874-07

**Authors:** 


### CONTENTS:

### ORIGINAL COMMUNICATIONS.

Article I.—Protoplasm.—Its Relation to Vitality. (Continued.) By Prof. H. R. Noel, M. D. .......... 97

Article II.—Care of Children's Teeth between the ages of Six and Fifteen. By Dr. P. E. White .......... 105

Article III.—The Use of Alcohol in Medical Practice. (Concluded.) By John Morris M. D. .......... 113

Article IV.—American Dental Convention .......... 120

### SELECTED ARTICLES.

Article V.—Abnormal Mucous Membrane. By Dr. S. B. Tizzard .......... 121

Article VI.—Separating Teeth. By W. G. A. Bonwell, D. D. S .......... 129

Article VII.—Causes of Decay of Teeth .......... 133

Article VIII.—New Operation for Cleft Palate .......... 135

### EDITORIAL, ETC.

The Recent Decision in the Vulcanite Suit .......... 136

Annual Meetings of the Associations—

Southern Dental Association .......... 138

American Dental Association .......... 139

American Dental Convention .......... 139

American Academy of Dental Science .......... 139

American Dental Society of Europe .......... 139

### OBITUARY.

Dr. T. B. Hamlin .......... 140

### MONTHLY SUMMARY.

Neuralgia Treated by Phosphorus .......... 141

Chloral Poisoning Cured by Strychnia .......... 142

Blue Pus .......... 143

How to Check Coughs .......... 144

Determination of the Age of the Human Embryo by the Evolution of the Teeth .......... 144

Oxygen Gas as a Remedy in Disease .......... 144

